# Transcriptional Alterations by Ischaemic Postconditioning in a Pig Infarction Model: Impact on Microvascular Protection

**DOI:** 10.3390/ijms20020344

**Published:** 2019-01-15

**Authors:** Dominika Lukovic, Alfred Gugerell, Katrin Zlabinger, Johannes Winkler, Noemi Pavo, Tamás Baranyai, Zoltán Giricz, Zoltán V. Varga, Martin Riesenhuber, Andreas Spannbauer, Denise Traxler, András Jakab, Rita Garamvölgyi, Örs Petnehazy, Dietmar Pils, Levente Tóth, Rainer Schulz, Péter Ferdinandy, Mariann Gyöngyösi

**Affiliations:** 1 Department of Cardiology, Medical University of Vienna, A-1090 Vienna, Austria; alfred.gugerell@meduniwien.ac.at (A.G.); katrin.zlabinger@meduniwien.ac.at (K.Z.); johannes.winkler@meduniwien.ac.at (J.W.); noemi.pavo@meduniwien.ac.at (N.P.); martin.riesenhuber@meduniwien.ac.at (M.R.); andreas.spannbauer@meduniwien.ac.at (A.S.); denise.traxler-weidenauer@meduniwien.ac.at (D.T.); mariann.gyongyosi@meduniwien.ac.at (M.G.); 2 Department of Pharmacology and Pharmacotherapy, Semmelweis University, 1085 Budapest, Hungary; baranyai.tamas@med.semmelweis-univ.hu (T.B.); giricz.zoltan@med.semmelweis-univ.hu (Z.G.); varga.zoltan@med.semmelweis-univ.hu (Z.V.V.); 3 Department of Biomedical Imaging and Image-Guided Therapy, Medical University of Vienna, A-1090 Vienna, Austria; andras.jakab@kispi.uzh.ch; 4 Center for MR-Research, University Children’s Hospital, 8032 Zurich, Switzerland; 5 Institute of Diagnostic Imaging and Radiation Oncology, University of Kaposvár, 7400 Kaposvár, Hungary; garamvolgyi.rita@sic.ke.hu (R.G.); petnehazy.ors@sic.ke.hu (Ö.P.); 6 Center for Medical Statistics, Informatics, and Intelligent Systems, Medical University of Vienna, A-1090 Vienna, Austria; dietmar.pils@meduniwien.ac.at; 7 Department of Radiology, University of Pécs, 7624 Pécs, Hungary; toth.l.levente@gmail.com; 8 Institute of Physiology, Justus Liebig University Giessen, 35392 Giessen, Germany; rainer.schulz@physiologie.med.uni-giessen.de; 9 Pharmahungary Group, Graphisoft Park, 7 Záhony Street, H-1031 Budapest, Hungary; peter.ferdinandy@pharmahungary.com

**Keywords:** ischemia-reperfusion injury, acute myocardial infarction, ischemic postconditioning, transcriptome, porcine model

## Abstract

Although the application of cardioprotective ischaemia/reperfusion (I/R) stimuli after myocardial infarction (MI) is a promising concept for salvaging the myocardium, translation to a clinical scenario has not fulfilled expectations. We have previously shown that in pigs, ischaemic postconditioning (IPostC) reduces myocardial oedema and microvascular obstruction (MVO), without influencing myocardial infarct size. In the present study, we analyzed the mechanisms underlying the IPostC-induced microvascular protection by transcriptomic analysis, followed by pathway analysis. Closed-chest reperfused MI was induced by 90 min percutaneous balloon occlusion of the left anterior descending coronary artery, followed by balloon deflation in anaesthetised pigs. Animals were randomised to IPostC (*n* = 8), MI (non-conditioned, *n* = 8), or Control (sham-operated, *n* = 4) groups. After three hours or three days follow-up, myocardial tissue samples were harvested and subjected to RNA-seq analysis. Although the transcriptome analysis revealed similar expression between IPostC and MI in transcripts involved in cardioprotective pathways, we identified gene expression changes responding to IPostC at the three days follow-up. Focal adhesion signaling, downregulated genes participating in cardiomyopathy and activation of blood cells may have critical consequences for microvascular protection. Specific analyses of the gene subsets enriched in the endothelium of the infarcted area, revealed strong deregulation of transcriptional functional clusters, DNA processing, replication and repair, cell proliferation, and focal adhesion, suggesting sustentative function in the endothelial cell layer protection and integrity. The spatial and time-dependent transcriptome analysis of porcine myocardium supports a protective effect of IPostC on coronary microvasculature post-MI.

## 1. Introduction

Since the first demonstration of cardioprotection in a postconditioned cat model, decades of intensive research have been devoted to the translation of ischaemic postconditioning (IPostC) to clinical settings [1]. Preclinical research on rodents argues in favor of IPostC-elicited cardioprotection manifesting improved outcomes of myocardial infarction (MI) [2,3]. However, large animal models of IPostC have demonstrated varying success with regards to improvement in left ventricular function and reduced infarct size [4,5]. Recent clinical studies have reported heterogeneous outcomes when applying IPostC immediately after achieving reperfusion in primary percutaneous coronary intervention for ST-segment elevation myocardial infarction (STEMI) [6,7,8]. The recent DANAMI-3 IPost and POST trials failed to show any benefit of postconditioning for patients in regards to all-cause death and heart failure [6,9]. The discrepancies between preclinical models showing beneficial effects, and clinical trials failing to show meaningful cardioprotection, have been attributed to differences in study design, the assessment of cardioprotective effects (imaging or biochemical markers vs. clinical outcome), and heterogeneous study populations in clinical trials with an impact of co-morbidities and co-medications, particularly anaesthetics [10].

We have previously shown that performing six 30 s cycles of ischaemia/reperfusion (I/R) stimuli applied immediately after a prolonged ischaemic insult in a clinically relevant porcine model of reperfused MI, fails to attenuate the size of myocardial necrosis assessed by cardiac magnetic resonance imaging (MRI). However, IPostC conferred cardioprotection on the coronary microvasculature and significantly reduced myocardial oedema and microvascular obstruction (MVO), which were quantified by late gadolinium-enhanced cardiac MRI [11]. MVO is a no-reflow phenomenon after reperfusion therapy in acute MI, and a major contributor to the reperfusion injury. MVO is characterized as a prolonged perfusion defect caused by endothelial cell swelling and protrusion, with accumulation of platelets, and neutrophils resulting in capillary obstruction. It is recognized as a serious complication for coronary flow restoration, and a poor prognostic marker of infarct expansion and left ventricular remodeling [12]. The occurrence and extent of the MVO on late enhancement gadolinium MRI images detected 3–5 days after primary percutaneous coronary intervention in patients with STEMI has recently been shown to be a strong predictor of long-term cardiovascular adverse events [13].

As shown in detail previously, MVO was detected and quantified three days post MI using cardiac MRI [11]. Briefly, the hypointense zone in the center of hyperenhancement in late gadolinium-enhanced MRI images was quantified, and values were expressed relative to the left ventricular mass. IPostC significantly decreased the MVO volume compared to myocardial infarction without cardioprotection [11].

Therefore, the aim of the present study was to identify molecular targets and mechanisms that explain the microvasculature protection elicited by IPostC. We performed transcriptomic analyses on myocardial samples isolated from pigs that underwent MI followed by IPostC. The results were compared to the transcriptomic changes in animals after non-conditioned MI, as unbiased RNA-sequencing enables the identification of novel targets [10,14,15]. Our previous analysis of gene expression patterns in the infarcted and remote (non-ischaemic) areas in pigs that had underwent repetitive I/R stimuli without infarction, revealed the capacity of preconditioning cycles to induce intrinsic remote conditioning of the myocardial regions unaffected by ischaemia, suggesting the prevention of adverse ventricular remodeling [16]. Conditioning-induced myocardial salvage is a bi-phasic action; we therefore evaluated the transcriptomic profile of the infarct-related and remote tissues at three hours and three days after MI and IPostC.

To the best of our knowledge, this is the first study to evaluate the effect of IPostC on microvascular protection at the molecular level, at different time windows, in a translational large animal model of MI.

## 2. Results

### 2.1. Assessment of Infarct Severity

Detailed data, including cardiac function and assessment of MVO for these experimental groups were published previously [11].

Briefly, IPostC failed to reduce the area at risk as assessed by cardiac imaging and necrosis in triphenyl tetrazolium chloride (TTC) histological staining, but reduced the extent of both myocardial oedema and MVO. Analysis of circulating biochemical markers demonstrated an effect of MI and IPostC on the enzymatic release of myoglobin, an early marker of myocardial damage, and cardiac troponin I type 3 (cTnI3), an indicator of acute myocardial injury with typical release within hours up to a few days after MI.

**Figure 1:**
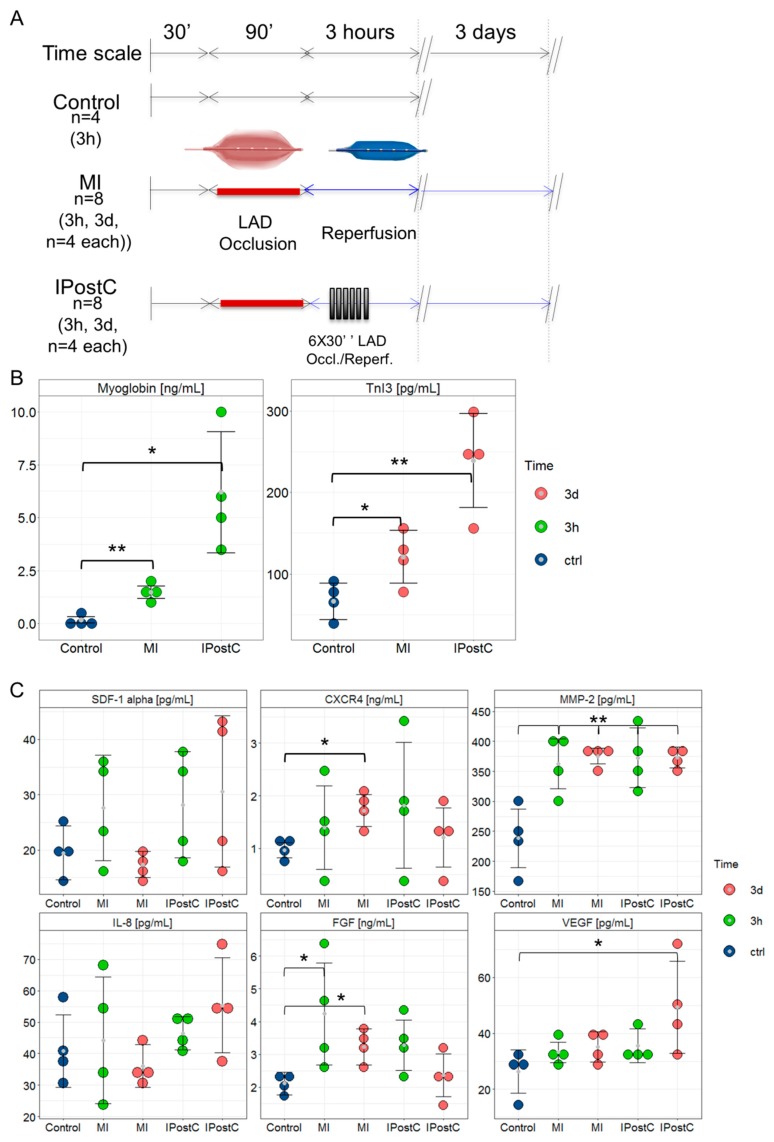
Study design for the porcine closed-chest reperfused model of myocardial infarction and ELISA results. Pigs underwent 90-min occlusion of the mid-left anterior descending coronary artery (LAD), followed by reperfusion. Postconditioning stimuli were induced at the onset of the reperfusion phase by six 30 s cycles of ischaemia/reperfusion. (**A**) Infarct severity was assessed by myoglobin plasma concentration and cardiac troponin I 3 serum concentrations. (**B**) Circulating cardioprotective markers in the Control (*n* = 4), MI-3h (*n* = 4), MI-3d (*n* = 4), IPostC-3h (*n* = 4), and IPostC-3d (*n* = 4) groups. (**C**) * *p* < 0.05; and ** *p* < 0.01 (one-way ANOVA/Tukey post-hoc).

MI with (*p* = 0.0243) and without (*p* = 0.0016) IPostC induced a significant release of myoglobin into the plasma compared to the control group at the 3-h follow-up, and increased serum cTnI3 concentrations at the 3-day follow-up (without IPostC, *p* = 0.0004; with IPostC, *p* = 0.0061; Figure 1B), without any differences among groups.

### 2.2. Circulating Cardioprotective Markers

Figure 1C demonstrates the release of potentially cardioprotective circulating proteins following MI and IPostC. SDF-1α and IL-8 plasma concentrations in the MI-3h, MI-3d, IPostC-3h, and IPostC-3d groups remained unchanged compared to the control group. The release of CXCR4 into plasma was significantly increased compared to controls only in the MI-3d group (*p* = 0.0048). MI and IPostC induced a parallel significant increase in MMP-2 in the plasma at three hours (MI-3h: *p* = 0.007; IPostC-3h: *p* = 0.011) and three days (MI-3d: *p* = 0.0004; IPostC-3d: *p* = 0.0003) post-infarction compared to the control group. FGF plasma concentrations were significantly elevated only in the MI-3h (*p* = 0.0409) and MI-3d (*p* = 0.0384) groups. In contrast, a significant increase in VEGF was observed at 3d in the IPostC (*p* = 0.0384) group.

### 2.3. Analysis of Next Generation Sequencing Expression Data

All sequenced genes were subjected to principal component analysis (PCA) to identify clustering, revealing a distinct difference between sham-operated control and treatment groups. However, considerable variation in the population structure was found between the IPostC and MI groups (Supplementary Figure S1).

**Figure 2:**
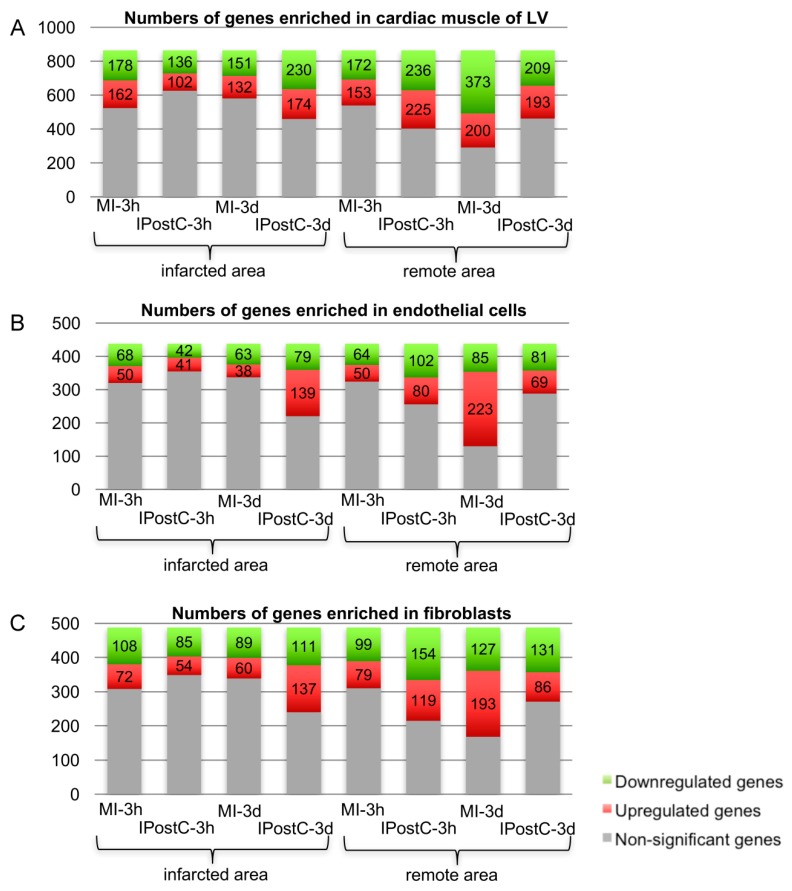
Functional clustering of genes. High-throughput screening of significantly regulated genes enriched in cardiac muscle (**A**), endothelial cells (**B**), and fibroblasts (**C**).

In order to assess the transcriptional response in individual cell types present within the myocardial tissue, we used bioinformatic analysis to classify gene transcripts according to their documented specific enrichment in cardiac muscle cells, endothelial cells, and fibroblasts (Figure 2A–C). Comparison across treatment groups showed that considerable changes in gene expression were evident, particularly at the later time point in the remote area of both the MI and IPostC groups. In the infarcted area at three days, a higher number of transcripts were dysregulated in all cell types, including the endothelium, and a substantial transcriptomic response to MI, with or without IPostC, was observed in the remote area of the IPostC-3d and MI-3d groups (Supplementary Figures S2 and S3).

Differentially expressed genes (DEGs) were clustered according to their involvement in molecular pathways and networks by performing Signalling Pathway Impact Analysis (SPIA) with Kyoto Encyclopedia of Genes and Genomes (KEGG) data. We found largely analogous enrichment of calcium signaling and smooth muscle cell contraction in both MI and IPostC groups in the first window, and an inhibition of apoptosis-associated genes in the MI group but not IPostC group (Supplementary Table S2).

Three days post intervention, a more complex transcriptomic response (Supplementary Table S3) was detected with activation of immune responses and upregulation of transcripts involved in cell stress. Interestingly, the infarcted area was more strongly influenced in the IPostC group, whereas the remote myocardium was impacted in the MI group.

### 2.4. Effect of IPostC on Pro-Survival Pathways

**Figure 3:**
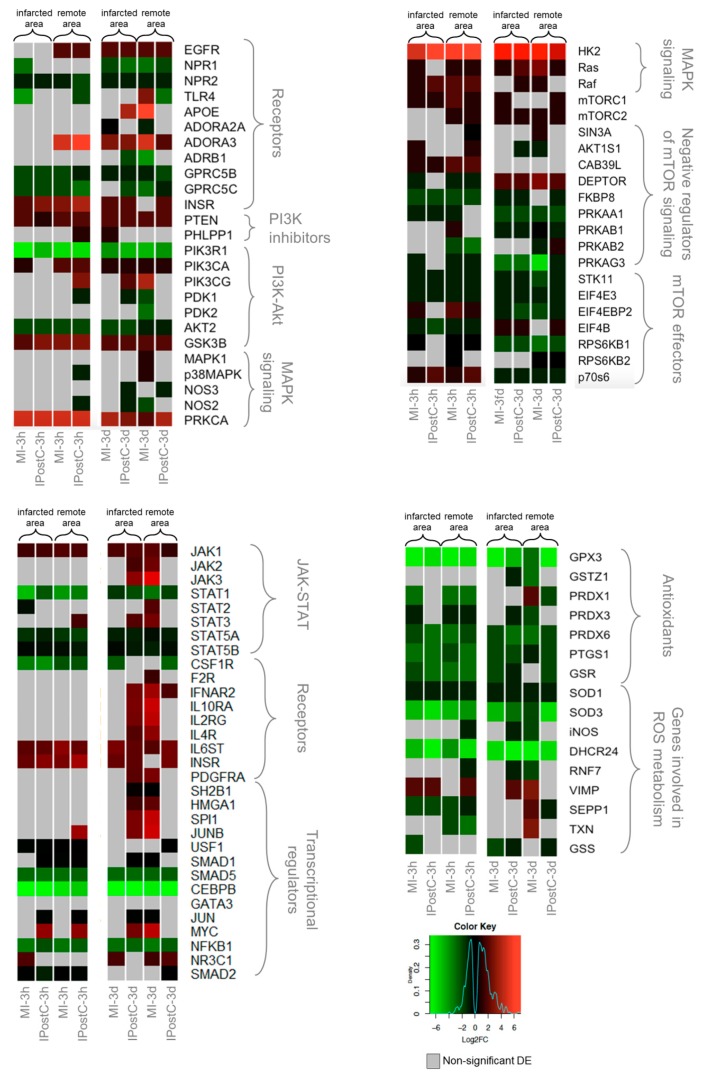
Heat maps of selected differentially expressed genes (DEGs) involved in cardioprotection, showing regional and time-dependent changes in expression changes. DEGs in the MI-3h (*n* = 3), MI-3d (*n* = 3), IPostC-3h (*n* = 3), and IPostC-3d (*n* = 3) groups are related to the control group (*n* = 3). Colors indicate the extent of log2 fold decrease (green) or increase (red) (*p*-value < 0.05, moderated *t*-statistics adjusted for multiple testing). Grey indicates non-significant regulation.

We directly compared IPostC and MI to discern cardioprotective mechanisms induced by IPostC from the more general transcriptional response induced by ischaemia and reperfusion. Figure 3 and supplementary Table S4 show the log2 fold changes in genes relative to the control group that are involved in cardioprotective pathways.

We found a generally similar response between the MI and IPostC groups in the remote and infarcted zones of both time windows. However, some genes exhibited distinct regulation by IPostC. The expression of several receptors (EGFR, ADORA3, and INSR) was significantly elevated in both the MI and IPostC groups. Kinases responsible for signal transduction exhibited a similar expression pattern in the MI and IPostC groups.

The negative mTOR regulator, DEPTOR, was significantly overexpressed in all groups at three days, together with inhibition of mTOR effectors. An evident activation of JAK/STAT signaling, based on the expression of major pathway components, was not confirmed in the pigs, and the expression of transcriptional regulators associated with JAK/STAT was ambiguous. However, many genes involved in reactive oxygen species (ROS) metabolism and genes encoding antioxidants were downregulated, and exhibited similar regulation across all sample groups in the AMI and IPostC groups.

### 2.5. Analysis of Genes with Opposite Regulation in MI and IPostC

**Figure 4:**
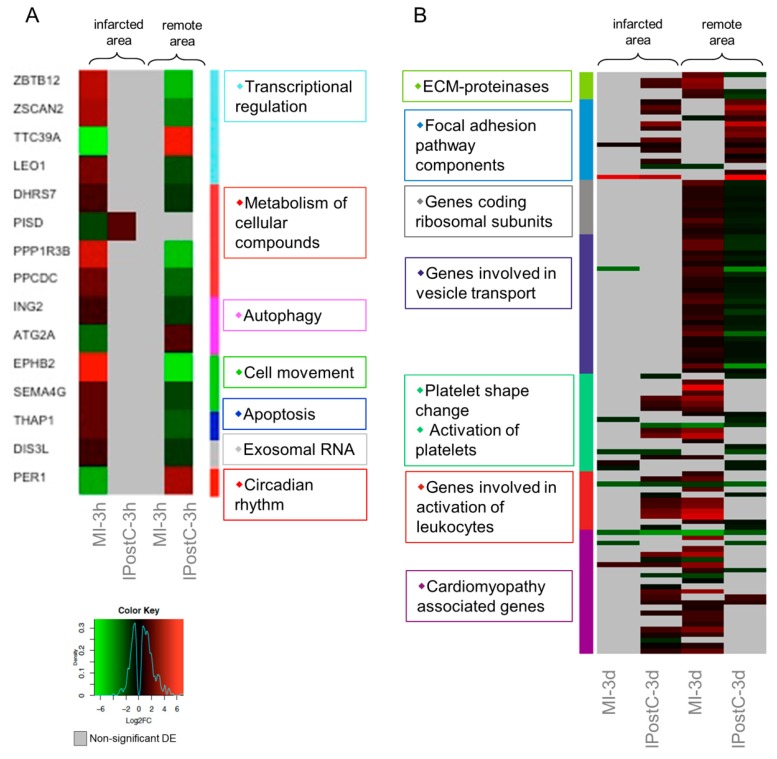
Heat maps of differentially expressed genes (DEGs) with opposite regulation in the myocardial infarction (MI) (**A**), and ischaemic postconditioning (IPostC) groups (**B**). DEGs in the MI-3h (*n* = 3), MI-3d (*n* = 3), IPostC-3h (*n* = 3), and IPostC-3d (*n* = 3) groups are related to the control group (*n* = 3). Colors indicate the extent of log2 fold decrease (green) or increase (red) (*p*-value < 0.05, moderated *t*-statistics adjusted for multiple testing). Grey indicates non-significant regulation.

A total of 15 transcripts exhibited opposite regulation between the MI and IPostC groups at the three hour follow-up (Figure 4A), suggesting that IPostC causes only minor transcriptional changes in the early window. In contrast, the second window revealed more than 150 genes with significant opposite regulation. Clustering based on pathway enrichment (Figure 4B) indicated downregulation of genes responsible for extracellular matrix (ECM) degradation (MMP14, and MMP9), and upregulation of the focal adhesion pathway (ZYX, CAVs, and LAMB). The remote area inhibited transcription of ribosomal proteins in the IPostC group, but not the MI group, suggesting increased protein synthesis after MI that was attenuated by IPostC. Similarly, gene sets participating in the formation and transport of cellular vesicles (VPS, DNM, KDELR, and SCAMPs) were significantly downregulated in the remote area of the IPostC group, but induced in the MI group.

In addition, we observed downregulation of transcripts involved in the activation of platelets and leukocytes (SERPINE, SELE, VCAM1, ICAM2, L1CAM, and CCLs) in the remote area of the IPostC group compared to the MI group. Similar gene expression patterns were observed in the cluster of cardiomyopathy genes (GNAs, MTHFR, CREBs, and FASN).

Taken together, the results suggest that IPostC triggers a downregulation of ECM-proteinases, ribosomal subunits, platelet and leukocyte adhesion, and vesicular transport proteins, and it also induces focal adhesions. In addition, IPostC inhibits the activation of inflammatory leukocytes and cardiac hypertrophy on the expression level.

### 2.6. Role of Focal Adhesion Signalling, Regulation of Cell Volume, and Microvascular Protection in IPostC

Upregulation of the focal adhesion pathway is associated with beneficial mechanical and functional changes in the cardiac tissue involving cell structure and volume, resulting in the observed protection against myocardial oedema and MVO [11]. A detailed analysis showed that subunits of integrin receptors (ITGB1, ITGB2, initiating focal adhesion signaling) were significantly upregulated in the infarcted zone of the IPostC group.

Overexpression of cytoskeletal proteins responsible for the regulation of focal adhesion was evident in both the remote and infarcted zones of the IPostC-3d group. Tyrosine kinases involved in the activation and transduction of focal adhesion signals were significantly upregulated.

**Figure 5:**
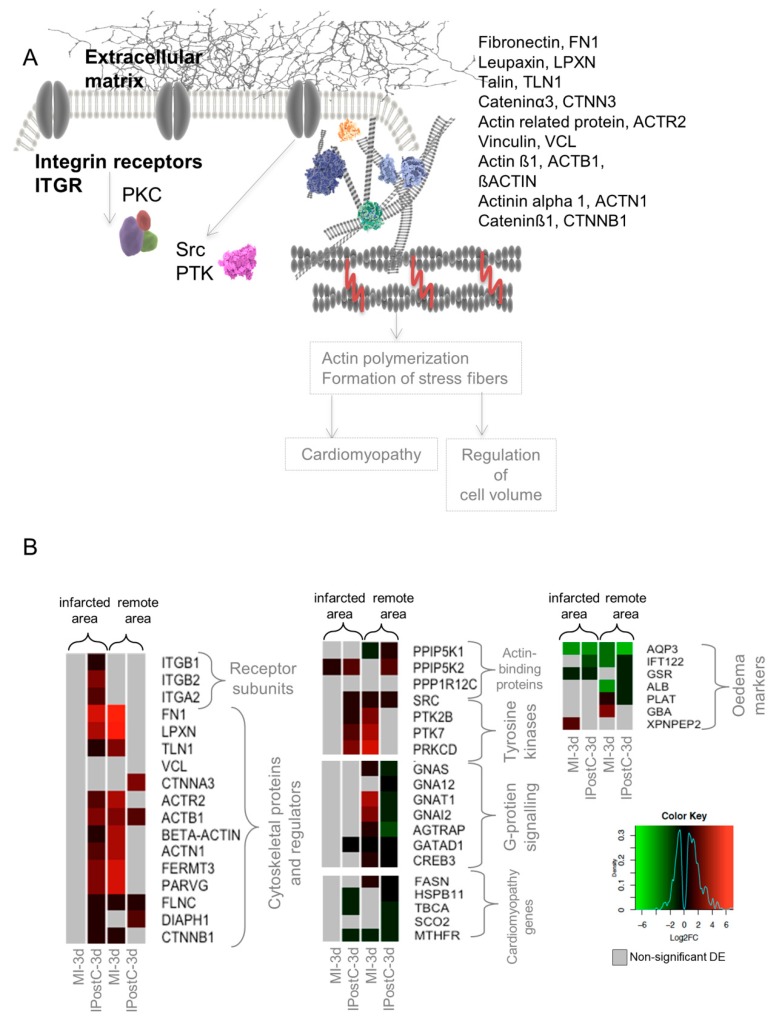
Simplified scheme for the focal adhesion pathway (**A**), and heat maps representing differentially expressed genes (DEGs) involved in the given pathway (**B**). DEGs in the MI-3h (*n* = 3), MI-3d (*n* = 3), IPostC-3h (*n* = 3), and IPostC-3d (*n* = 3) groups are related to the control group (*n* = 3). Colors indicate the extent of log2 fold decrease (green) or increase (red) (*p*-value < 0.05, moderated *t*-statistics adjusted for multiple testing). Grey indicates non-significant regulation.

We hypothesized that focal adhesion activation and changes in cell structure affected the expression of genes involved in cardiac muscle hypertrophy (cardiomyopathy) and the regulation of cell volume (formation of oedema) (Figure 5A). We observed significant downregulation of transcripts involved in cardiomyopathy in the remote and infarcted zones of the IPostC group. Similarly, the expression of oedema markers was downregulated or non-significantly regulated. Changes in the expression of oedema markers in the MI groups were ambiguous (Figure 5B).

**Figure 6:**
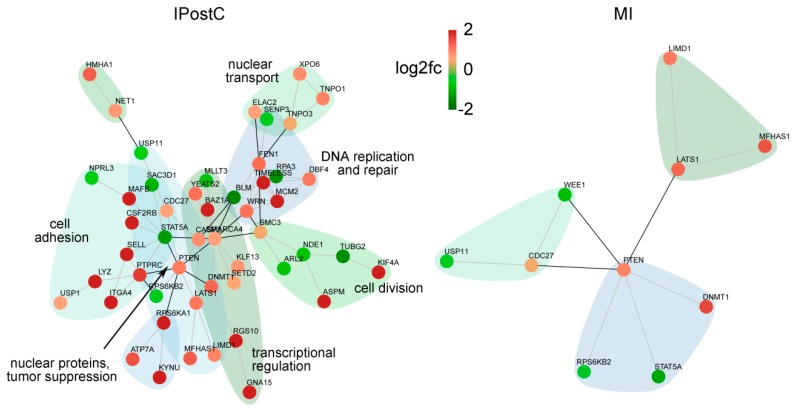
Networks of protein–protein interactions of deregulated transcripts with high enrichment scores in endothelial cells. DEGs in the infarcted zones at three days were clustered according to the string database of protein–protein interactions. Red nodes indicate higher, and green nodes lower expression in IPostC compared to controls. Groups with more than five members are annotated according to their main function.

We specifically analyzed the subset of genes enriched in the endothelium of the infarcted area for investigating the mechanism of microvascular protection. Clustering according to interactions and function (Figure 6) showed a strong impact on transcriptional dysregulation, DNA processing, replication and repair, cell proliferation, and adhesion in the endothelium. Factors involved in general cell adhesion and cytokine receptor signaling, such as DBF4, ITGA4, and LIMD1, and more specifically in leukocyte adhesion (SELL, PTPRC) were upregulated in the infarcted area of the IPostC group, suggesting sustentative function in the endothelial cell layer protection and integrity.

### 2.7. qPCR Validation of Transcripts Involved in Focal Adhesion

**Figure 7:**
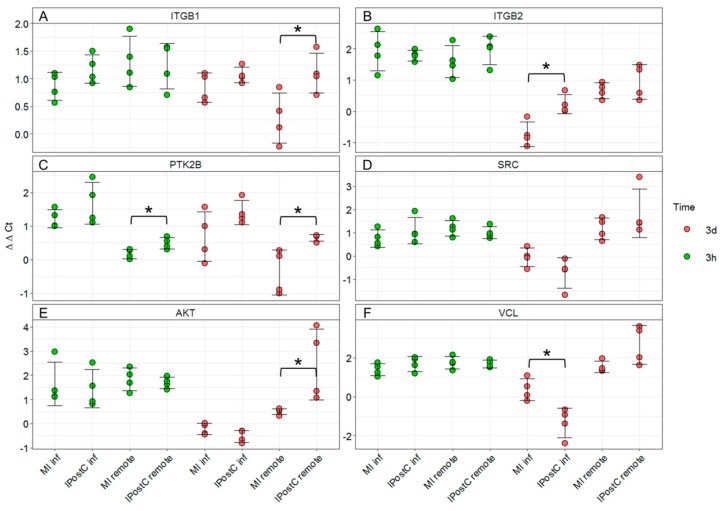
mRNA changes in gene transcripts involved in focal adhesion signaling measured by quantitative PCR. Expression of integrin subunit ß-1 (ITGB1) (**A**), integrin subunit ß-2 (ITGB2) (**B**), protein tyrosine kinase 2-ß (PTK2B) (**C**), SRC proto-oncogene (SRC) (**D**), V-Akt murine thymoma viral oncogene homolog 1 (AKT) (**E**), and vinculin (VCL) (**F**). * *p* < 0.05; between MI-3h (*n* = 4)/IPostC-3h (*n* = 4) and MI-3d (*n* = 4)/IPostC-3d (*n* = 4) in the infarcted and remote areas (one-way ANOVA/Tukey post-hoc).

After identifying individual DEGs involved in the focal adhesion pathway, we validated these changes using qPCR analysis. Normalized gene expression revealed analogous gene regulation by IPostC compared to MI, confirming RNA-seq data (Figure 7). Subunits of integrin receptors ITGB1 (*p* = 0.03) and ITGB2 (*p* = 0.01), were upregulated in the IPostC-3d group (Figure 7A,B). Protein tyrosine-kinase 2-beta (PTK2B) was upregulated in the remote area of the IPostC group at both early (*p* = 0.03) and late time points (*p* = 0.05; Figure 7C). The proto-oncogene non-receptor tyrosine kinase SRC was not significantly regulated by IPostC (Figure 7D).

mRNA expression of v-akt murine thymoma viral oncogene homolog 1 (AKT) was significantly increased in the remote area of the IPostC-3d group (*p* = 0.05; Figure 7E). Vinculin (VCL) expression was significantly decreased in the infarcted area of the IPostC-3d group, and a trend towards a significant increase was observed in the remote area of the IPostC-3d group (*p* = 0.06; Figure 7F).

## 3. Discussion

To the best of our knowledge, this is the first description of myocardial whole-transcriptome expression patterns after postconditioning stimuli in a translational experimental model, demonstrating time-dependent changes of gene expression in remote and ischaemic regions. The transcriptional profile of genes involved in cardioprotective pathways exhibited largely analogous responses after MI and IPostC. However, we show a substantial transcriptional response to IPostC after three days. IPostC induced differential regulation of transcripts coding for ECM proteinases, genes involved in focal adhesion, cellular metabolism, and protein biosynthesis and processing. Although IPostC cycles mediated certain changes, they were apparently insufficient for translation into a clinically relevant cardioprotective effect. The obvious beneficial effect of IPostC in the pig model is a protection of the coronary microvasculature, reducing oedema and MVO [11]. In line with these findings, we identified several molecular targets involved in microvascular protection induced by IPostC.

### 3.1. Large Animal Model of IPostC

Na et al. published the first study to introduce the concept of IPostC. They described prevention of reperfusion-induced ventricular fibrillation by postconditioning stimuli in a cat model [1]. Although the subsequent experimental studies in rodents [3], rabbits [17], and pigs [4] provided a substantial amount of data, a clear and universal beneficial effect could not be confirmed due to the varying outcomes in different experimental settings. In our study, we utilized a reperfused porcine AMI model with 90 min coronary occlusion representing myocardial ischaemia, followed by reperfusion. This is the best available preclinical model to simulate human primary percutaneous coronary intervention (PCI) [18]. Postconditioning cycles were applied immediately at the onset of reperfusion, as several studies have demonstrated a diminished beneficial effect of IPostC when I/R cycles are applied in the later phase of reperfusion [17,19]. We showed that six 30 s I/R stimuli applied immediately after achieving reperfusion are ineffective for reducing enzymatically estimated infarct sizes at three hours or three days. Similarly, left ventricular ejection fraction and myocardial necrosis were not affected by IPostC [11], and the protective effect is limited to a reduction of microvascular oedema and MVO.

### 3.2. Release of Circulating Cardioprotective Cytokines

Salvaging affected myocardial tissue after MI is attributed to the secretion of cytokines and inhibition of other pro-inflammatory stimuli. Preconditioning stimuli have been shown to enhance the enzymatic release of chemotactic cytokines with parallel mobilization of hematopoietic stem cells [20,21], which may have a protective effect against I/R injury at the myocyte level.

We observed a significant increase in MMP-2 and VEGF in response to MI, without an impact of IPostC, confirming earlier reports [22]. Increased VEGF may contribute to cardiac microvascular protection, but VEGF expression was not significantly induced in myocardial tissue samples by IPostC compared to MI at the examined time points. The release of SDF-1, CXCR4, IL-8, and FGF in the MI and IPostC groups exhibited a tendency towards an increase, but this did not reach significance. The plasma VEGF concentration significantly increased three days after IPostC. Studies demonstrating the benefits of preconditioning stimuli in pig [21] and rat [20] models detected the release of cardioprotective cytokines within a few hours following the initiation of reperfusion. Insufficient secretion of cardioprotective cytokines into the peripheral circulation may explain the failure of IPostC to confer a protective benefit in this study.

### 3.3. Analogous Response of MI and IPostC to Prosurvival Pathways

Numerous studies have reported the activation of cellular prosurvival pathways in response to postconditioning stimuli. PI3K/AKT [23], RISK [24], JAK/STAT [25], and mTOR signaling [2] are thought to play a prominent role in conditioning-elicited cardioprotection.

Our study indicates that IPostC and MI enforce the regulation of genes involved in cardioprotective pathways (RISK, JAK/STAT, and mTOR) in a similar manner independent of the time window and myocardial region. This finding contradicts other reports showing postconditioning-mediated activation of prosurvival cascades. In postconditioned male Wistar rat hearts, PI4K has a significant role in opening the mitochondrial pore, thus providing cardioprotection [2]. The delayed protective effect of IPostC has been shown to persist during activation of the PI3K-Akt pathway [23]. Further studies performed on rodent animals have confirmed an association of IPostC-mediated cardioprotection with the activation of JAK/STAT [26]. Zhang et al. demonstrated that IPostC reduces oxidative injury in rats [3]. Our results demonstrate largely unchanged expression of genes involved in ROS metabolism and oxidative stress, but an increase in ROS cannot be excluded. The discrepancies may be related to the particular animal model and study protocol. Furthermore, data from recent clinical trials have not provided convincing results of IPostC-elicited cardioprotection [6,27].

### 3.4. Opposite Regulation of Genes Involved in MI with/without IPostC, Demonstrating the Beneficial Effect of IPostC in Microvascular Protection and Tissue Oedema

The number of significantly de-regulated genes between the IPostC and MI groups in infarcted and remote areas at three hours was limited. In contrast, at three days more than 150 significantly DEGs were detected, representing a complex transcriptional response to IPostC. Apparently, the eventual effects in the first window of protection are not caused by transcriptional regulation, but rather by alterations on the protein or cellular level.

Downregulation of ECM proteinases and upregulation of focal adhesion components in the remote area of the IPostC-3d group indicated that postconditioning stimuli induce cytoskeleton-based signaling. Upregulation of the focal adhesion pathway has been attributed to cardioprotective preconditioning stimuli in mice [28] and perfused hearts [29]; however, upregulation of focal adhesion transcripts in the IPostC model has not been shown thus far. The focal adhesion pathway regulates mechanical and functional changes in cardiac tissue inducing alterations in cellular structure, proliferation rate, and cell migration [30], which may play a role in reducing microvascular oedema and MVO. The spatial differences in overexpression of distinct focal adhesion pathway components (e.g., integrins, cytoskeletal proteins in the infarcted area, and tyrosine kinases in the remote area) suggest an interplay between the remote and infarcted zones of the myocardium. In addition, we recently showed that a porcine model of preconditioning exhibits substantial gene expression changes in the remote area, implying the prevention of adverse remodeling effects [14].

We show that a large cluster of genes involved in the formation of ribosomal subunits and vesicular transport is downregulated in the remote area of the IPostC-3d group compared to upregulated expression in the MI-3d group. A recent proteomics study in pigs demonstrated a decrease in these protein clusters until 24 h after MI, followed by induction of the same proteins over the next few days as a sign of gradual activation of the healing process and fibrosis [31]. Our data indicate that IPostC delays the onset of remodeling; however, for a definite conclusion, more time-dependent investigations will be necessary.

Our data also suggest that IPostC downregulates genes participating in the activation and adhesion of platelets and leukocytes. This provides a functional link to the inhibition of MVO and cellular oedema in this IPostC model [11]. MVO is associated with increased adhesion and aggregation of platelets, neutrophils, and leukocytes to the ischaemia-affected tissues, contributing to the microcirculatory perfusion deficit [32]. Here, we demonstrate that the genes responsible for the activation of inflammatory leukocytes were significantly downregulated in the remote area of the IPostC-3d group, suggesting that attenuated MVO by IPostC is mediated by transcriptional changes.

The more profound impact on endothelial gene regulation in the infarcted area by IPostC at three days, both in terms of the number of altered transcripts (Figure 2) and functional clustering (Figure 6), indicates a significant contribution of the microvasculature. An overall induction of transcripts for DNA replication and processing, regulation of transcription, and cell division and adhesion may explain the molecular mechanism of IPostC-mediated microvascular protection.

Two clinical studies have observed that the IPostC intervention procedure has the capacity to increase coronary blood flow [33] and inhibit endothelial injury [34] in AMI patients. These findings point towards a potent inhibitory effect of IPostC on microcirculatory disturbances.

### 3.5. Limitations

Baseline cTnI3 and myoglobin measurements to determine the enzymatic infarct size for the individual treatment groups were not assessed, assuming normal values at baseline in the animals. Even a preconditioning stimulus with three cycles of 10 min ischaemic/reperfusion did not result in elevation of cTnI3 and myoglobin in our previous experiments [16].

Analysis of the transcriptome provided a comprehensive comparison of time-dependent and regional changes in the expression of genes between the MI and IPostC groups; however, the sample size was limited and included three biological replicates each in the MI, IPostC, and sham-operated control groups.

Myocardial tissue consists of heterogeneous cells, mostly cardiomyocytes, endothelial cells, and fibroblasts. In order to avoid the transcriptomic analysis of minor cells (e.g., neurons), we selected transcripts typical for cardiac cells by bioinformatic enrichment. This selection avoids overestimation of genetic profiles of cells that are presumably playing a marginal role in heart conditioning. The myocardial tissue used in transcriptomic analysis represents heterogeneous starting material, and thus, the results do not provide insight into individual gene expression changes on distinct cellular levels.

Inclusion of an additional three days follow-up for a sham-operated control group would promote a more corrected comparison between the three days follow-up conditioning and sham groups. However, we expect constant gene expression in the animals without MI, with the same anaesthetic protocol, before tissue harvesting of the sham animals. Using the above-described study design, we have reduced the number of sham animals, which was requested by the animal ethics committee. The efficacy of cardioprotection by IPostC was assessed only at two time-points, therefore we cannot rule out any beneficial or adverse effects on the later outcomes.

Isoflurane was applied in all groups and like other anaesthetics, may confer cardioprotection [35], and thus may complicate the identification of IPostC effects. This may explain why no reduction in infarct size was detected, but we aimed to adhere to a protocol with high relevance to clinical settings.

## 4. Materials and Methods

### 4.1. Study Design

Large animal experiments were conducted in accordance with the Guide for the Care and Use of Laboratory Animals published by the US National Institutes of Health (NIH publication No. 85-23, revised 2011), and were approved by the Ethics Committee of the Hungarian National Food Chain Safety Office (Approval number: SOI/31/26-11/2014). Experiments and results were reported according to the ARRIVE guidelines [36]. The study design is shown in Figure 1A. The detailed experimental protocol has been published previously [11].

Briefly, female domestic pigs (*n* = 20, 25–30 kg) were sedated by subcutaneous administration of 12 mg/kg ketamine hydrochloride, 1 mg/kg xylazine, and 0.04 mg/kg atropine after overnight fasting. Following endotracheal intubation, the anaesthesia was deepened with 1.5–2.5 vol % isoflurane, 1.6–1.8 vol % O_2_, and 0.5 vol % N_2_O. A 6F introduction sheath (Terumo Medical Corporation, Somerset, NJ, USA) was inserted into the right femoral artery. Following administration of 200 IU/kg of heparin, 6F right coronary guiding catheters were introduced into the left and right coronary ostium to perform selective angiography of the left and right coronary arteries.

To induce MI, the LAD, after the second diagonal branch, was occluded by the inflation of a coronary angioplasty balloon (2.75 mm diameter, 15 mm length; Maverick, Boston Natick, MA, USA) at 5 atm for 90 min. Control angiography was performed to confirm occlusion of the artery. Intracoronary injection of 5000 IU heparin was administered after occlusion, and immediately before reperfusion, to avoid clot formation at the occlusion site. Reperfusion was initiated via balloon deflation. The pigs were randomized to IPostC (*n* = 8) or non-conditioned MI (*n* = 8) groups, while four animals underwent sham operations (Control, *n* = 4). Animals in the IPostC and MI groups were further randomized to a follow-up of either three hours (IPostC-3h group, *n* = 4; MI-3h group, *n* = 4) or three days (IPostC-3d group, *n* = 4; MI-3d group, *n* = 4; Figure 1A). Postconditioning was elicited by six cycles of 30 s occlusion/reperfusion of the LAD. Selective coronary angiography of the left coronary artery was performed immediately after re-opening the LAD to confirm reperfusion, and also immediately after each postconditioning I/R cycle. At the pre-defined follow-up, animals were humanely euthanized under general anaesthesia induced by subcutaneous injection of 12 mg/kg ketamine hydrochloride, 1 mg/kg xylazine, and 0.04 mg/kg atropine with an intravenous injection of 10% potassium chloride solution.

Blood and tissue samples were collected at indicated time points and subjected to downstream analysis. The explanted hearts were placed in ice-cold saline, and small parts (approx. 1 cm^3^) of the distal anterior infarcted area (below the origin of the second diagonal branch, appearing as macroscopic haemorrhagia with oedema at three hours or pale-grey color at three days) and the remote, visually normal posterior wall were collected. TTC staining of the remaining myocardium confirmed the correct sampling locations [11].

### 4.2. ELISA

The expression of cardiac enzymes was assessed by commercially available ELISA kits, according to the manufacturer´s instructions. Myoglobin (Porcine Myoglobin ELISA kit, USCN, Wuhan, China) was measured from plasma samples collected at the three hours follow-up. The serum level of cTnI3 (Porcine cTnI3 ELISA kit, USCN, Wuhan, China) was assessed at the three days follow-up.

The concentrations of circulating cardioprotective markers were assessed according to the manufacturer’s protocols. The concentrations of stromal-derived factor 1-alpha (SDF-1α; Porcine SDF-1α ELISA kit, Neoscientific, Langenfeld, Germany), chemokine C-X-C motif receptor 4 (CXCR4; Pig CXCR4 ELISA kit, Abbexa, Cambridge, UK), interleukin-8 (IL-8, Pig IL-8 ELISA kit, Abcam, Cambridge, UK), matrix metalloproteinase 2 (72 kDa MMP-2, Pig MMP-2 ELISA kit, MyBiosource, San Diego, CA, USA), fibroblast growth factor (FGF, pig FGF ELISA kit, Neoscientific, Langenfeld, Germany), and vascular endothelial growth factor (VEGF, pig VEGF ELISA kit, Neoscientific, Langenfeld, Germany) were determined by spectrophotometric readouts on a VICTOR3 plate reader (Perkin Elmer, Waltham, MA, USA).

### 4.3. RNA Extraction

Tissues from the non-infarcted (remote zone, e.g., from the posterior myocardial region) and infarcted areas of the left ventricle were collected from explanted hearts, washed with PBS (Sigma-Aldrich, St. Louis, MO, USA), cut into pieces, immediately transferred into cryotubes containing 1.2 mL RNAlater^®^ (Ambion, Austin, TX, USA), and stored at −80 °C. Tissue slices (~30 mg) were homogenized using Precellys lysing kit CK28 beads (VWR, Vienna, Austria) in Qiazol lysis buffer (Qiagen, Hilden, Germany). Total RNA was isolated using the RNeasy Microarray^®^Tissue kit (Qiagen, Hilden, Germany).

### 4.4. RNA Sequencing

The total RNA fractions of the myocardial samples were subjected to mRNA deep sequencing using the Illumina (San Diego, CA, USA) platform. The NEBNext Poly(A) mRNA Magnetic Isolation Module (New England Biolabs, Ipswich, MA, USA) was utilized for mRNA fragmentation and enrichment. Fragmented and primed mRNA was reverse transcribed to cDNA. The cDNA library was synthesized and enriched using the NEBNext Ultra Directional RNA Library Kit (New England Biolabs, Ipswich, MA, USA). Sequencing was performed on the HiSeq 2500 platform to a mean depth of 15–20 million paired-end reads per sample at the Core Facility of the Medical University of Vienna.

### 4.5. Quantitative PCR (qPCR) Analysis for Validation of Pre-Selected Genes

mRNA (1 μg) was isolated from myocardial probes, followed by reverse transcription to cDNA using the QuantiTect^®^Reverse Transcription kit (Qiagen, Hilden, Germany). Target gene expression was assessed by qPCR on an Applied Biosystems 7500 Real-Time PCR System (Life Technologies, Thermo Fisher Scientific, Waltham, MA, USA) using a QuantiTect SYBR Green Reaction kit (Qiagen, Hilden, Germany), according to the manufacturer´s instructions, in biological and technical replicates. Genes of interest were selected based on significantly differential expression in the RNA-seq analysis, and their involvement in the focal adhesion pathway. The primers (Supplementary Table S1) for the targeted porcine cDNAs were designed using Primer3 software and purchased from Microsynth (Balgach, Switzerland).

Target gene expression rates were normalized to the geometric means of three reference genes: ACTB, HPRT1, and PPIA. The relative gene expression was calculated using the ΔΔCt method. Expression changes were calculated relative to myocardial expression levels in the control animals.

### 4.6. Statistical Analysis

Continuous parameters were compared using one-way ANOVA with multiple comparisons for the mean at each time point using Tukey HSD post-hoc tests (R software, R Foundation for Statistical Computing, Vienna, Austria). Details of the biostatistical analyses are described in the Supplementary Methods. Gene expression in the ischaemic and remote myocardium of the MI and IPostC groups at three hours and three days was compared to expression in the non-ischaemic myocardium of the control group. The concentrations calculated from absorbance measurements in ELISA experiments were obtained by applying a four-parameter logistic curve to the standard measurements. *p* < 0.05 was considered significant.

## 5. Conclusions

In summary, the present study is a comprehensive analysis of IPostC, taking into account the capacity to induce microvascular protection by reducing obstruction and oedema, and to show whole transcriptome changes. We identified relevant transcriptional changes in genes with opposite regulation in the MI and IPostC groups, which are involved in the activation of focal adhesion, translational processes, and blood cells that may have critical consequences in response to I/R injury, even if these transcriptomic changes do not translate into clinically relevant infarct size reduction at the assessed time points.

## Supplementary Materials

Supplementary Materials can be found at http://www.mdpi.com/1422-0067/20/2/344/s1.

## Abbreviations

DEG	differentially expressed gene
ECM	extracellular matrix
I/R	ischaemia/reperfusion
IPostC	ischaemic postconditioning
LAD	left anterior descending artery
MI	myocardial infarction
MRI	magnetic resonance imaging
MVO	microvascular obstruction
PCA	principal component analysis
SPIA	signaling pathway impact analysis
STEMI	ST-segment elevation myocardial infarction
TTC	triphenyl tetrazolium chloride
