# Supplementary material for: Transcriptional Alterations by Ischaemic Postconditioning in a Pig Infarction Model: Impact on Microvascular Protection
Source: Int J Mol Sci. 2019 Jan 15;20(2):344. doi: 10.3390/ijms20020344 (PMC6358966; doi:10.3390/ijms20020344)
Supplement: Supplementary file 1 [file ijms-20-00344-s001.zip › ijms-408385-proofreading done SI.pdf]

Transcriptional alterations by ischaemic postconditioning in a pig infarction model: impact on microvascular protection

Dominika Lukovic, Alfred Gugerell, Katrin Zlabinger, Johannes Winkler, Noemi Pavo, Tamás Baranyai, Zoltán Giricz, Zoltán V. Varga, Martin Riesenhuber, Andreas Spannbauer, Denise Traxler, András Jakab, Rita Garamvölgyi, Örs Petnehazy, Dietmar Pils, Levente Tóth, Rainer Schulz, Péter Ferdinandy, Mariann Gyöngyösi

Supplementary Information

Supplementary results

Gene	Name	5´ → 3´	Sequence	GC [%]	Amplicon [bp]
ITGB1	Integrin ß-1	forward	GGGACGTGTTGGGAGACATT	55	123
		reverse	CCGCAGACACACTCTCCATT	55	
PTK2B	Protein tyrosine kinase 2ß	forward	CAGCAGACGTTCCAGCAGTA	55	108
		reverse	ACAGCGATAGGTCTCCTGGT	55	
SRC	Non-receptor tyrosine kinase	forward	AATGCGGAGAACCCAAGAGG	55	80
		reverse	TCGGACACAGAGAGGCAGTA	55	
PIK3CA	Phosphatidylinositol 3-Kinase, Catalytic Subunit α	forward	AAAGGCCGAAAGGGTGCTAA	50	127
		reverse	ACCCATGAGGTACAGGCCAA	55	
AKT3	V-Akt Murine Thymoma Viral Oncogene Homolog 3	forward	TGATGAGGACGGAATGGACTG	52	70
		reverse	TGCAGAGTAGGAAAACTGAGG	50	
VCL	Vinculin	forward	CGTCCGGGTTGGAAAAGAGA	55	130
		reverse	TGACTGAAGCATCTGGGCTG	55	
RHOA	Ras Homolog Family Member- A	forward	CCCAATGTGCCCATCATCCT	55	102
		reverse	TGGTTTTACTGGCTCCTGCT	50	
ACTB	Actin ß	forward	TCAACACCCCAGCCATGTAC	55	107
		reverse	CTCCGGAGTCCATCACGATG	60	
PPIA	Peptidylprolyl Isomerase A	forward	GTCTTCTTCGACATCGCCGT	55	120
		reverse	TCCTTTCTCCCCAGTGCTCA	55	
HPRT1	Hypoxanthine Phosphoribosyltransferas1	forward	CCCAGCGTCGTGATTAGTGA	55	131
		reverse	ATCTCGAGCAAGCCGTTCAG	55	

Supplementary Table 1. Primers used for qPCR

3 hours							
	**group**	**KEGG ID**	**Pathway**	**Size**	**z**	**pG**	**Status**
infarcted area	MI	4210	Apoptosis	46	16	0.0086	Inhibited
	IPostC	3320	PPAR signaling pathway	31	9	0.0530	Activated
	MI +IPostC	4020	Calcium signaling pathway	70	17	0.0224	Activated
		4270	Vascular smooth muscle contraction	51	13	0.0004	Activated
remote area	MI	4210	Apoptosis	46	13	0.0189	Inhibited
	MI +IPostC	4270	Vascular smooth muscle contraction	36	20	0.0468	Activated
		4020	Calcium signaling pathway	70	27	0.0200	Activated

Supplementary Table 2. Signaling Pathway Impact Analysis of DEGs in infarcted area of first window

3 days								
	group	KEGG ID	Pathway		Size	NDE	pG	Status
infarcted area	MI	4270	Vascular smooth muscle contraction		51	15	0.0034	Activated
	IPostC	4620	Toll-like receptor signaling pathway	A common denominator of induced immune processes was the upregulation of genes implicating defences against autologous cells undergoing various forms of stress.	51	25	0.0001	Activated
		4650	Natural killer cell mediated cytotoxicity		47	25	0.0002	Activated
		4062	Chemokine signaling pathway		80	38	0.0006	Activated
		4664	Fc epsilon RI signaling pathway		25	13	0.0017	Activated
		4666	Fc gamma R-mediated phagocytosis		43	20	0.0017	Activated
		4670	Leukocyte transendothelial migration		60	25	0.0037	Activated
		4662	B cell receptor signaling pathway		30	18	0.0259	Activated
		4014	Ras signaling pathway		105	47	0.0287	Activated
		4810	Regulation of actin cytoskeleton		88	35	0.0395	Activated
		3320	PPAR signaling pathway		31	9	0.0530	Activated
remote area	MI	4650	Natural killer cell mediated cytotoxicity	A common denominator of induced immune processes was the upregulation of genes implicating defences against autologous cells undergoing various forms of stress.	47	37	2.51E-06	Activated
		4670	Leukocyte transendothelial migration		60	44	1.11E-05	Activated
		4664	Fc epsilon RI signaling pathway		25	20	5.07E-04	Activated
		4620	Toll-like receptor signaling pathway		51	32	0.0010	Activated
		4062	Chemokine signaling pathway		80	53	0.0031	Activated
		5010	Mitochondrial dysfunction		108	90	0.0046	Activated
		5414	Dilated cardiomyopathy		47	36	0.0365	Inhibited
		4066	HIF-1 signaling pathway		55	43	0.0481	Activated
	IPostC	4270	Vascular smooth muscle contraction		51	20	0.0066	Activated

Supplementary Table 3. Signaling Pathway Impact Analysis of DEGs in infarcted area of first window

Differentially expressed genes, DEG; Kyoto encyclopedia of genes and genomes (KEGG) pathway ID, KEGG ID; number of differentially expressed genes, NDE; global p-value, pG

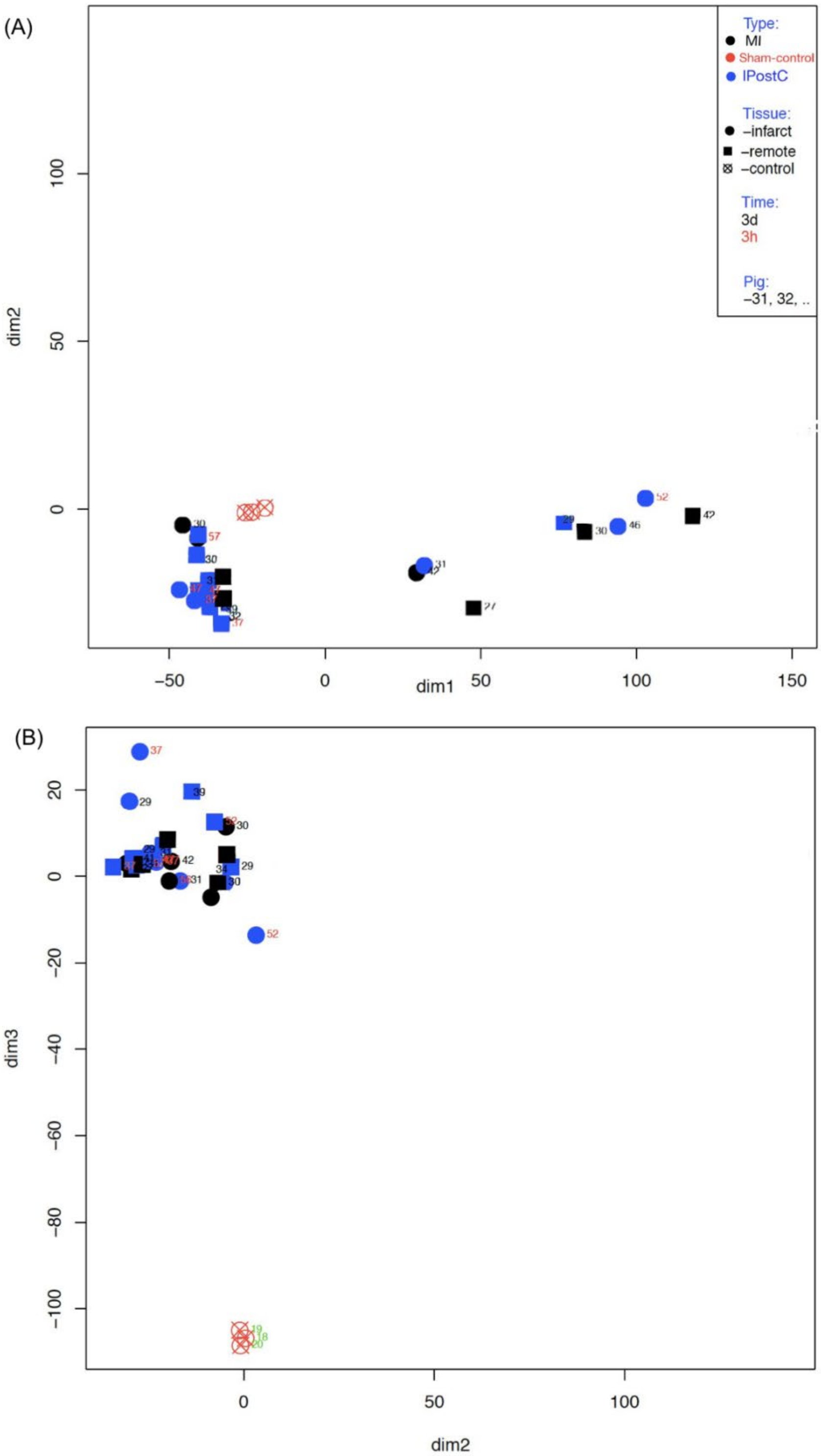

Supplementary Figure 1. Global (Principal component analysis, PCA).

The first (dim1) and the second (dim2) dimensions of the PCA **(A),** the second (dim2) and the third (dim3) dimensions of the PCA **(B)**

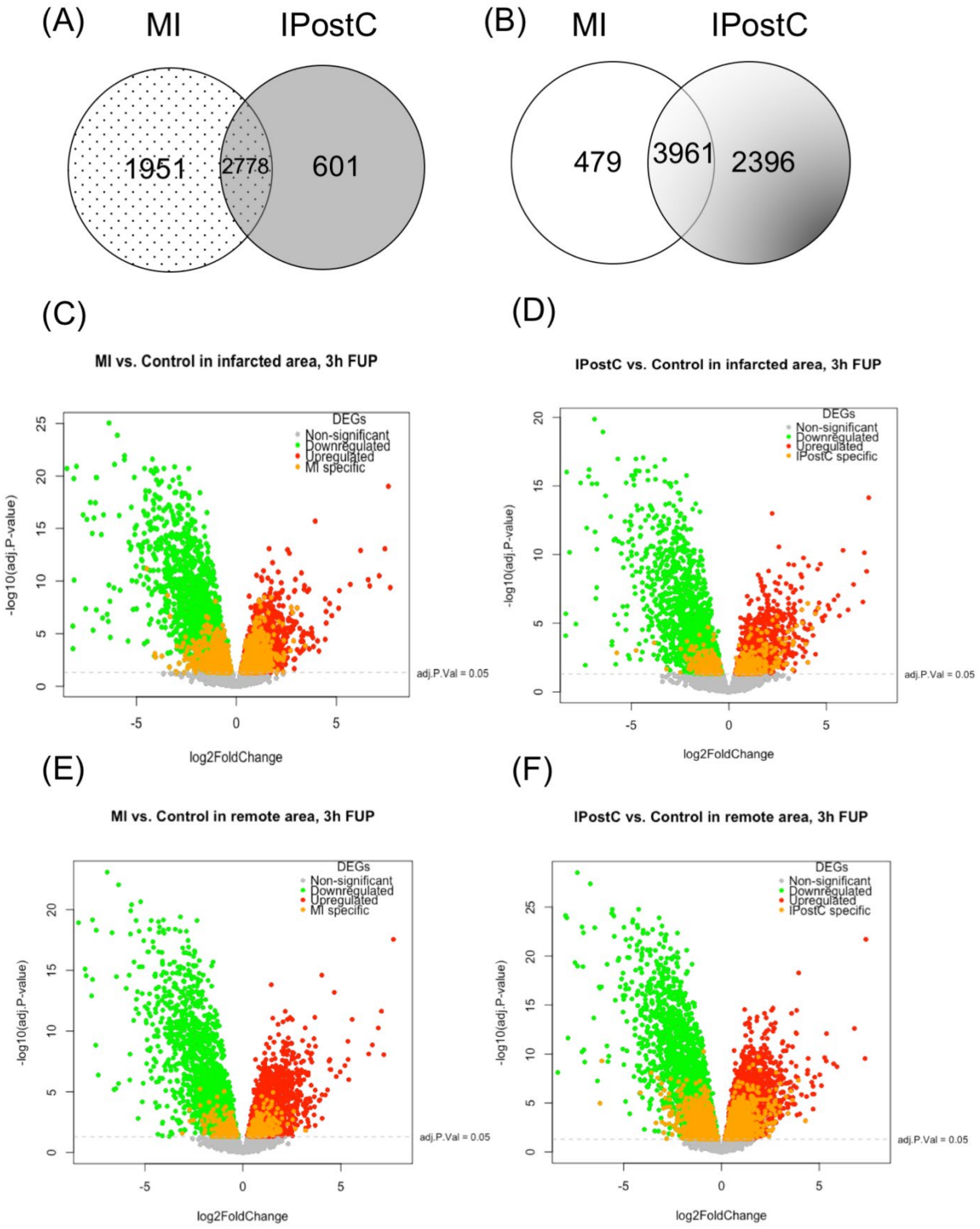

Supplementary Figure 2 Analysis of differentially expressed genes (DEGs) of infarcted area and remote area in the first window

(A) Venn diagram showing the comparison of differentially regulated genes between myocardial infarction (MI, n=3) and ischemic postconditioning (IPostC, n=3) group in infarcted area at three hours follow-up.

(B) Venn diagram showing the comparison of differentially regulated genes between myocardial infarction (MI, n=3) and ischemic postconditioning (IPostC, n=3) group in remote area at three hours follow-up.

(C) Volcano plot for DEGs in infarcted area of MI group (n=3) after three hours follow-up compared to the control group.

(D) Volcano plot for DEGs in infarcted area of IPostC group (n=3) after three hours follow-up compared to the control group.

(E) Volcano plot for DEGs in remote area of MI group (n=3) after three hours follow-up compared to the control group.

(F) Volcano plot for DEGs in remote area of IPostC group (n=3) after three hours follow- up compared to the control group.

Volcano plot reporting adjusted P-value [−log10(adj. P. Val, y axis] as a function of log2 (fold change). Each point represents an individual transcript.

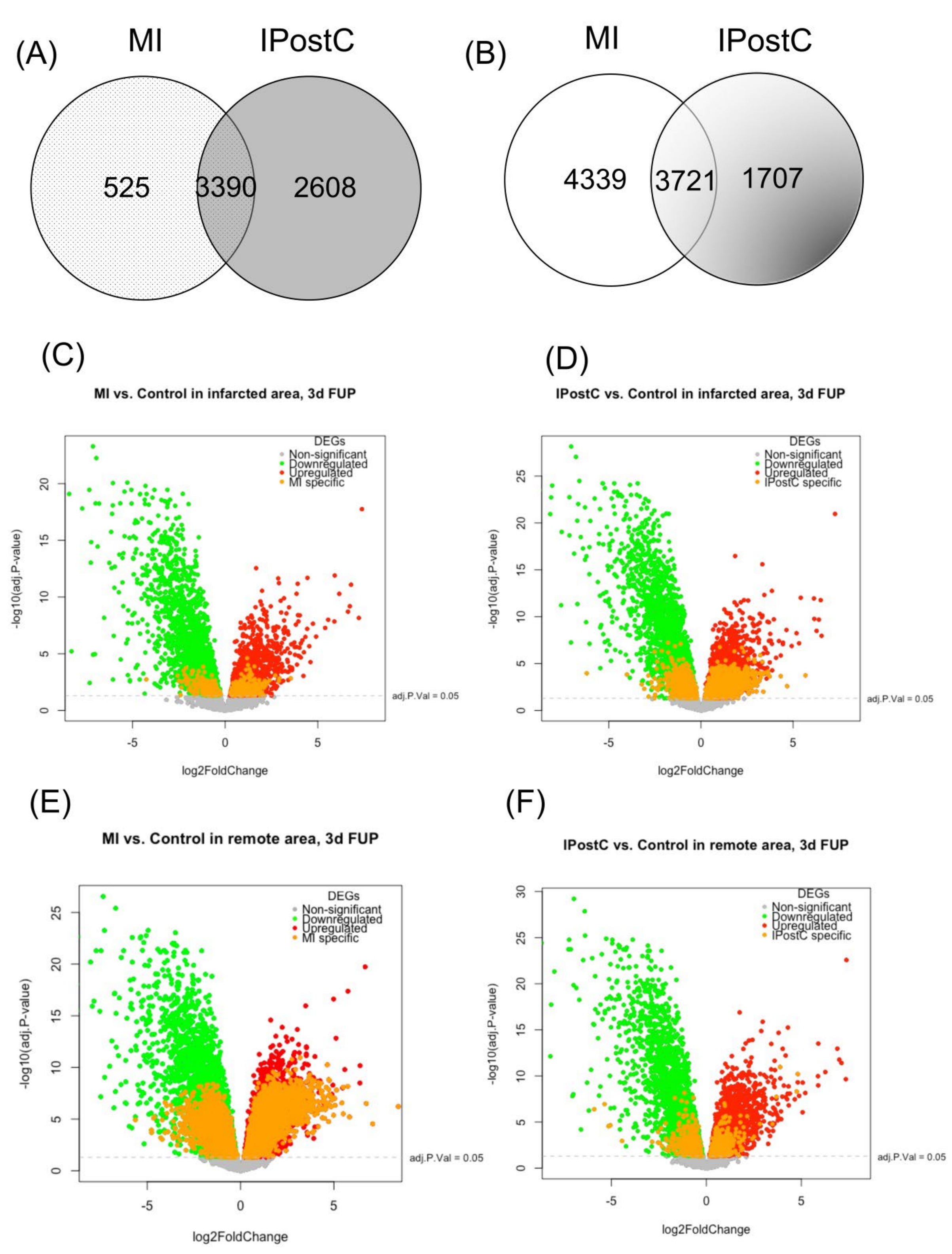

Supplementary Figure 3 Analysis of differentially expressed genes (DEGs) of infarcted area and remote area in the second window

(A) Venn diagram showing the comparison of differentially regulated genes between myocardial infarction (MI, n=3) and ischemic postconditioning (IPostC, n=3) group in infarcted area at three days follow-up.

(B) Venn diagram showing the comparison of differentially regulated genes between myocardial infarction (MI, n=3) and ischemic postconditioning (IPostC, n=3) group in remote area at three days follow-up.

(C) Volcano plot for DEGs in infarcted area of MI group (n=3) after three days follow-up compared to the control group.

(D) Volcano plot for DEGs in infarcted area of IPostC group (n=3) after three days follow-up compared to the control group.

(E) Volcano plot for DEGs in remote area of MI group (n=3) after three days follow-up compared to the control group.

(F) Volcano plot for DEGs in remote area of IPostC group (n=3) after three days follow- up compared to the control group.

Volcano plot reporting adjusted P-value [−log10(adj. P. Val, y axis] as a function of log2 (fold change). Each point represents an individual transcript.

Supplementary methods

Bioinformatic analysis

Analysis of samples was performed on the HiSeq2500 platform with a depth of 15-20 million paired-end reads per samples. Quality control and demultiplexing of reads was assessed by FastQC. Alignment to Sus scrofa genome (Sscrofa 10.2) was performed applying RNA-Seq Unified Mapper (v2.0.4) [1] .

HTSeq software was used to count reads into Sus scrofa gene model [2].

Downstream analysis and statistical calculations were performed on R Studio platform (version 3.2.3).

EdgeR package was utilised to identify differentially expressed genes (DEGs) and read count values were normalised and trimmed to remove genes with a low expression. Differently expressed genes were considered statistically significant at the threshold of FDR<0.05. Moderated t-statistics, F-statistics, and the log odds of differential expression were computed by empirical Bayes shrinkage of the standard errors towards a common value. False discovery rate (FDR) below 5% was accounted as statistically significant. FDR cut-off of 10% was considered for some contrasts, for relevant gene lists for biomedical interpretation. Unsupervised hierarchical cluster analysis was performed using the Euclidean distance as the distance function and the Ward algorithm in R, using centred and scaled log2 expression values. Principal Component Analysis (PCA) was conducted for centred and scaled values.

Genes with significant differential expression were annotated according to the function and subjected to signalling pathway impact analysis (SPIA) [3]. Deregulation of the single gene was represented by the $\log_2$ fold changes. Family-wise error rate (FWER) correction and false discovery rate (FDR) correction were applied to determine the regulation of genes and pathway topology with a cut-off of 5 %.

Gene Enrichment Profiler Database [4] and Protein Atlas Database [5] were used to identify genes with high enrichment score for specific cell types present in cardiac tissue. Protein networks were constructed using the R package dnet [6], based on protein-protein interactions supplied by the String database, version 10 [7]. Nodes shown were restricted to a maximum of 50.

Supplementary References

1. Grant GR, Farkas MH, Pizarro AD, Lahens NF, Schug J, Brunk BP, Stoeckert CJ, Hogenesch JB, Pierce EA. Comparative analysis of RNA-Seq alignment algorithms and the RNA-Seq unified mapper (RUM). *Bioinformatics* 2011;**27**:2518-2528.
2. Anders S, Pyl PT, Huber W. HTSeq--a Python framework to work with high-throughput sequencing data. *Bioinformatics* 2015;**31**:166-169.
3. Tarca AL, Draghici S, Khatri P, Hassan SS, Mittal P, Kim JS, Kim CJ, Kusanovic JP, Romero R. A novel signaling pathway impact analysis. *Bioinformatics* 2009;**25**:75-82.
4. Benita Y, Cao Z, Giallourakis C, Li C, Gardet A, Xavier RJ. Gene enrichment profiles reveal T-cell development, differentiation, and lineage-specific transcription factors including ZBTB25 as a novel NF-AT repressor. *Blood* 2010;**115**:5376-5384.
5. Uhlen M, Fagerberg L, Hallstrom BM, Lindskog C, Oksvold P, Mardinoglu A, Sivertsson A, Kampf C, Sjostedt E, Asplund A, Olsson I, Edlund K, Lundberg E, Navani S, Szigyarto CA, Odeberg J, Djureinovic D, Takanen JO, Hober S, Alm T, Edqvist PH, Berling H, Tegel H, Mulder J, Rockberg J, Nilsson P, Schwenk JM, Hamsten M, von Feilitzen K, Forsberg M, Persson L, Johansson F, Zwahlen M, von Heijne G, Nielsen J, Ponten F. Proteomics. Tissue-based map of the human proteome. *Science* 2015;**347**:1260419.
6. Fang H, Gough J. The `dnet' approach promotes emerging research on cancer patient survival. *Genome Medicine* 2014;**6**:64.
7. Szklarczyk D, Franceschini A, Wyder S, Forslund K, Heller D, Huerta-Cepas J, Simonovic M, Roth A, Santos A, Tsafou KP, Kuhn M, Bork P, Jensen LJ, von Mering C. STRING v10: protein–protein interaction networks, integrated over the tree of life. *Nucleic Acids Res* 2015;**43**:D447-D452.
